# First Case Report of Arrhythmogenic Right Ventricular Cardiomyopathy Showing Refractory Ventricular Tachycardia Induced by Thyroid Storm due to Graves' Disease

**DOI:** 10.1155/2022/6078148

**Published:** 2022-06-23

**Authors:** Masaki Matsubara, Tomohiro Tanaka, Akinori Wakamiya, Tamiko Tamanaha, Hisashi Makino, Tomonori Tanei, Takeshi Aiba, Kengo Kusano, Kiminori Hosoda

**Affiliations:** ^1^Division of Diabetes and Lipid Metabolism, National Cerebral and Cardiovascular Center, 6-1 Kishibe-Shimmachi, Suita, Osaka 564-8565, Japan; ^2^Department of Cardiovascular Medicine, National Cerebral and Cardiovascular Center, 6-1 Kishibe-Shimmachi, Suita, Osaka 564-8565, Japan; ^3^Department of Breast and Endocrine Surgery, Osaka University Graduate School of Medicine, 2-2-E10 Yamadaoka, Suita, Osaka 565-0871, Japan

## Abstract

A 48-year-old man who was diagnosed with arrhythmogenic right ventricular cardiomyopathy (ARVC) due to a plakophilin 2 gene mutation developed acute both-sided heart failure with rapid atrial fibrillation and was hospitalized. After admission, sustained ventricular tachycardia, which was refractory to antiarrhythmic agents, occurred repeatedly, and required electrical cardioversion. He was diagnosed with thyroid storm due to Graves' disease, and treatment for hyperthyroidism was initiated. After the treatment, lethal arrhythmia did not reoccur, and biventricular heart failure ameliorated. To our best knowledge, this is the first report in English of a patient with ARVC showing refractory arrhythmia induced by thyroid storm due to Graves' disease.

## 1. Introduction

Arrhythmogenic right ventricular cardiomyopathy (ARVC) is characterized by an enlargement and hypofunction of the right ventricle and arrhythmia of the right ventricular origin and can cause sudden death due to arrhythmia in juveniles [1]. Thus, early diagnosis and appropriate treatment are important. ARVC is caused by genetic factors, one of which is a plakophilin 2 gene (*PKP2*) mutation, which is mainly expressed in the epidermis and cardiomyocytes, including Purkinje fibers and constituted desmosomes. Moreover, the connections between cardiomyocytes are impaired by mutations of this protein, leading to weakened myocardial structure [1, 2].

Graves' disease is a common disease, and it can induce a life-threatening condition, namely, thyroid storm (TS) [3]. Regarding the cardiovascular system affected by thyrotoxicosis, high-output heart failure with decreased systemic vascular resistance is expected [4]. However, low-output heart failure related to dilated cardiomyopathy has been reported as an unusual type of cardiac complication, which is usually reversible with hyperthyroidism treatment [5].

We herein report the first case of a patient with ARVC showing refractory ventricular tachycardia (VT) induced by TS due to Graves' disease. Moreover, the patient suffered from biventricular heart failure. Hyperthyroidism treatment was initiated, and it proved to be really effective. No lethal arrhythmia recurred after the treatment, and both-sided heart failure improved as well.

## 2. Case Presentation

**Figure 1:**
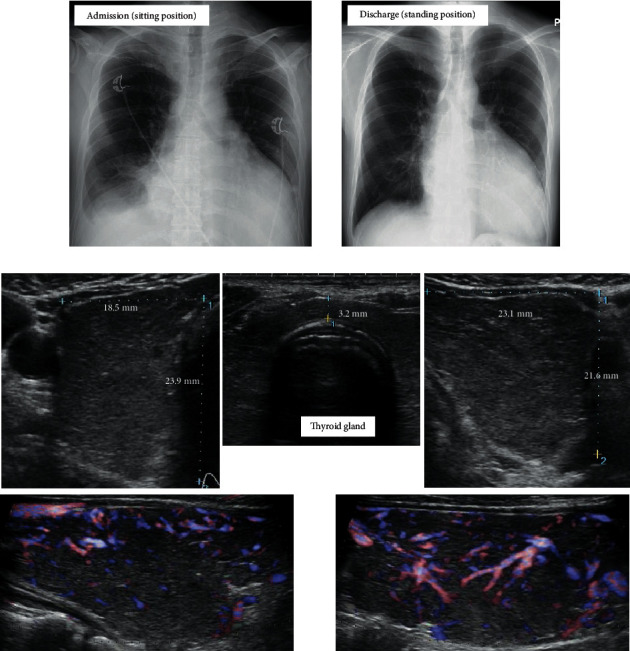
Imaging findings at first admission. (a) Chest X-ray on admission and discharge. (b) An ultrasonogram showing enlargement and increased blood flow of the thyroid gland.

**Figure 2:**
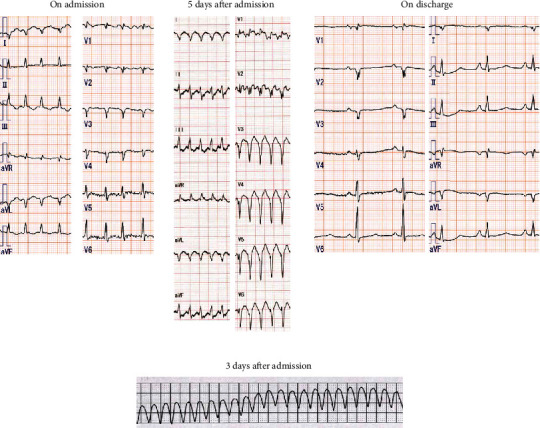
Electrocardiogram (ECG) findings at first admission. (a) Rapid atrial fibrillation on admission, atrial tachycardia on day 5, and normal sinus rhythm on discharge. (b) ECG monitor display showing ventricular tachycardia on day 3.

**Figure 3:**
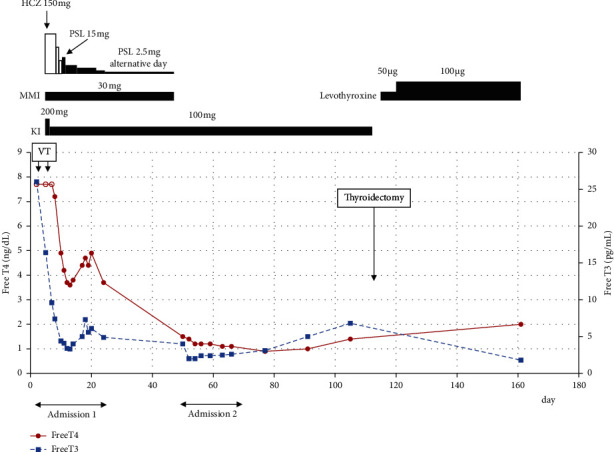
Total clinical course of the patient. Open circles indicate the value of free T4 mean more than 7.7 ng/dL. HCZ: hydrocortisone; PSL: prednisolone; MMI: methimazole; KI: potassium iodide; VT: ventricular tachycardia; T3: triiodothyronine; and T4: thyroxine.

The patient was a 48-year-old man diagnosed with ARVC due to PKP2 mutation [2] and constitutional jaundice. At the age of 35 years, catheter ablation of the right ventricular apical septum was performed for sustained VT with left bundle branch block-type and superior QRS axis. The thyroid function was euthyroid at the time; the thyroid-stimulating hormone (TSH) level was 1.53 (normal range, 0.5–5.5) *μ*U/mL, the free triiodothyronine (T3) level was 3.6 (normal range, 2.6–4.6) pg/mL, and the free thyroxine (T4) level was 1.5 (normal range, 1.1–1.8) ng/dL. Thereafter, under daily metoprolol intake of 80 mg, VT did not recur. He complained of palpitation six months before admission, and 80 mg/day sotalol was started. His laboratory data are shown in Table 1. Although palpitation was reduced, he had general fatigue and discontinued taking sotalol one month before consultation. He presented to our emergency department with worsening dyspnea, bilateral lower leg edema, a 5 kg weight gain, and watery diarrhea. As for vital signs, he was alert, his blood pressure was 123/87 mmHg, his heart rate was 150/min and irregular, his blood oxygen saturation was 95% (room air), and his body temperature was 37.7°C. Physical examination revealed jugular venous distention, coarse crackles in both lungs, pitting edema in the legs, and tremors of the upper limbs. Exophthalmos and ocular motility disturbances were not observed. A chest radiograph and electrocardiogram showed cardiac dilation, bilateral pleural effusion, and a rapid atrial fibrillation (AF) rhythm (Figures 1(a) and 2). Transthoracic echocardiography clarified dilation of the intravenous cava, right atrium, and right ventricle (RVDd = 57 mm), decreased right ventricular contractile function (TAPSE = 8 mm), compression of the left ventricle by the dilated right ventricle, and tricuspid regurgitation (peak pressure gradient = 15 mmHg) at the right side. LVDd and LVDs were 46 mm and 33 mm, respectively; however, they might have been underestimated owing to compression. Furthermore, a decreased ejection fraction (visual 30%) and absence of vegetation at the valves were observed. The plasma brain natriuretic peptide (BNP) and serum total bilirubin levels were elevated (Table 1). He was diagnosed with both-sided acute heart failure with rapid AF and admitted to the coronary care unit (CCU). The SOFA and APACHE II scores at admission were 2 and 8, respectively. He was given 80 mg/day intravenous furosemide and 0.25 mg intravenous digoxin, and heparinization was started. After admission, sustained VT occurred, so an electrical cardioversion was performed and intravenous amiodarone was administered (Figure 2). Moreover, atrial tachycardia, for which both 50 mg intravenous lidocaine and 20 milliequivalent magnesium sulfate were ineffective, occurred and required electrical cardioversion again, and 1 *μ*g/kg/min intravenous landiolol was initiated (Figure 2). Thereafter, VT reoccurred; thus, electrical cardioversion was reperformed, and intravenous landiolol was increased to 3 *μ*g/kg/min. Blood chemistry revealed suppressed TSH level (<0.01 *μ*U/mL) and elevated free T3 (16.4 pg/mL) and free T4 levels (>7.7 ng/dL), and the patient was referred for hyperthyroidism. Thyrotropin receptor antibody (TRAb) measured using the electrochemiluminescence assay method was 24.7 (normal range, <2.0) IU/L, and goiter with increased blood flow was confirmed by ultrasonography (Figure 1(b)). Hence, the patient was diagnosed with Graves' disease. The total bilirubin level increased to 5.6 mg/dL at that time. Thus, he fulfilled the following diagnostic criteria for TS proposed by the Japan Thyroid Association [3]: thyrotoxicosis, tachycardia, and gastrointestinal/hepatic manifestations, and the findings in this case correspond to TS2 (suspected TS). The Burch–Wartofsky Point Scale (BWPS) score, which was proposed in 1993 and had been widely used for the diagnosis of TS, of 45 and >45 is suggestive of TS [6]. Although the total bilirubin level of this patient could not be appropriately evaluated due to constitutional jaundice, BWPS was 60 even when the heading of “gastrointestinal-hepatic dysfunction” among the diagnostic criteria was underscored as “moderate” (if a patient showed jaundice, it was scored as “severe”). Therefore, he was diagnosed with TS, and treatment with 150 mg/day intravenous hydrocortisone, 30 mg/day oral methimazole (MMI), and 200 mg/day potassium iodide (KI) was initiated. After initial treatment of the TS, lethal arrhythmia including VT did not reoccur, and he returned to a normal sinus rhythm several days later (Figure 2). As thyroid function gradually decreased, intravenous hydrocortisone was tapered off and altered to oral prednisolone (PSL), and KI was decreased to 100 mg/day. Regarding his cardiac function, when he was referred for hyperthyroidism, the central venous pressure (CVP) decreased from 25 mmHg at admission to 17 mmHg, and compression of the left ventricle was substantially reduced. However, EF did not improve after the abovementioned therapy. Treatment for TS was started at this time, and the echocardiography performed 6 days after the hyperthyroid treatment showed improvement in contractile function and dilation of the left ventricle. The EF was 60%–65%, and the LVDds and LVDs were 42 mm and 28 mm, respectively. In addition, right ventricular dilation and function improved (RVDd = 52 mm; TAPSE = 11.7 mm). The thyroid function test results 6 days after the initiation of TS therapy were as follows: free T3 = 3.4 pg/mL and free T4 = 3.7 ng/dL (Figure 3). The free T4 level remained high, but the free T3 level returned to normal. Consequently, furosemide and landiolol were also tapered off and discontinued, and amiodarone was adjusted according to the serum concentration and shifted to oral administration. Although an implantable cardioverter-defibrillator placement was recommended for possibly recurrent VT [7], the patient declined. Finally, symptoms such as dyspnea, leg edema, and watery diarrhea improved, and his body weight reduced approximately 10 kg compared with admission data, so he was discharged on day 28. When he left the hospital, his medications were 2.5 mg PSL on alternate days, 30 mg/day MMI and 100 mg/day KI for hyperthyroidism, 10 mg/day apixaban for anticoagulation, 2.5 mg/day bisoprolol and 150 mg/day amiodarone for the prevention of arrhythmia, and 2.5 mg/day enalapril for heart failure. His blood test results at the time of discharge are shown in Table 1.

**Figure 4:**
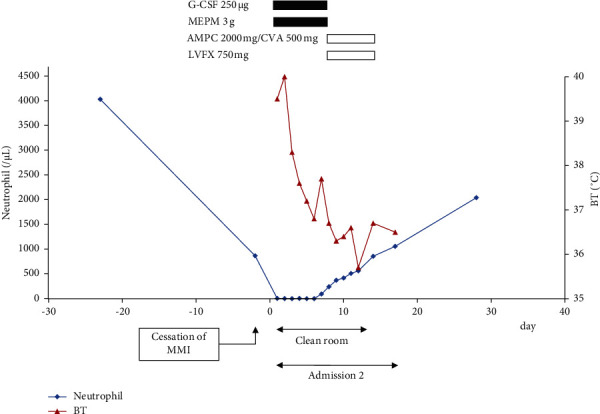
Clinical course of the patient during the second admission due to febrile neutropenia. G-CSF: granulocyte colony-stimulating factor; MEPM: meropenem; AMPC: amoxicillin; CVA: clavulanate; LVFX: levofloxacin; and BT: body temperature.

He became febrile and was transported to our hospital 22 days after discharge. His neutrophils decreased to 6/*μ*L and he was diagnosed with agranulocytosis due to MMI. He recovered after the initiation of empirical broad-spectrum antibiotics, granulocyte colony-stimulating factor (G-CSF), and acetaminophen treated in a clean room. Thyroid function was maintained by 100 mg/day KI and arrhythmia did not occur during the second admission. Additionally, some medications were changed because of drug-induced liver injury. The clinical course of second admission is shown in Figure 4. Total thyroidectomy was performed 48 days after the second discharge, and there were no complications, such as arrhythmia. Pathological observation showed that the resected thyroid was swelling slightly, and nodules were not detected. Hyperplasia of the large and small follicles and vacuolization where the epithelium contacted the colloid was observed. These findings were compatible with those of Graves' disease. After the operation, levothyroxine was initiated and adjusted according to the thyroid function. The total clinical course of the patient is shown in Figure 3.

## 3. Discussion

We herein report the first case of a patient with ARVC who presented with lethal arrhythmia, which was refractory to medication and electrical cardioversion induced by TS due to Graves' disease. Palpitation and decreased serum total cholesterol level were observed 6 months before the first hospitalization, and it is likely that he had already developed Graves' disease around this period. We considered that the cessation of sotalol might have triggered TS, which induced arrhythmia and led to the onset of biventricular heart failure. The treatment of TS was effective, succeeded to suppress refractory arrhythmias, and ameliorated both-sided heart failure. Thereafter, he developed agranulocytosis incidentally due to MMI. Finally, total thyroidectomy was performed without any complications and his thyroid function was controlled within the normal range by administering levothyroxine.

No previous English report describing a case complicated with ARVC and Graves' disease or the relationships between them has been published. Two cases of ARVC complicated with hyperthyroidism have been reported in Japanese. Although the cause of hyperthyroidism was not noted in both cases [8, 9], we considered they were complicated with Graves' disease since MMI was used for treatment. One case with ARVC complicated with hyperthyroidism repeated VT and required catheter ablation [8]. The other case was diagnosed with ARVC during treatment of hyperthyroidism and atrial tachycardia occurred when serum thyroid hormone levels elevated [9]. However, their clinical courses were not described in detail. Since PKP2 is rarely expressed in the thyroid gland [10], it is speculated that patients with ARVC harboring PKP2 mutation are not likely to develop thyroid diseases. As for Graves' disease, it is one of the autoimmune diseases induced by the production of autoantibodies to TSH receptors. Though mechanism of autoimmunity, namely, myocarditis, has been also implied in some ARVC patients [11], the association of Graves' disease and ARVC is unclear. Arrhythmia, especially AF, is a common complication of Graves' disease induced by activating the arrhythmogenic activity of the pulmonary veins [12]. Thus, when patients with ARVC are incidentally complicated with Graves' disease, hyperthyroidism can easily trigger arrhythmia. As for hyperthyroidism other than Graves' disease, amiodarone, which is often initiated for patients with ARVC to prevent arrhythmia, could develop thyrotoxicosis [13]. Previous reports have shown that patients with ARVC treated with amiodarone for long duration developed thyrotoxicosis due to destructive thyroiditis. Although corticosteroid was effective for controlling the thyroid function in some cases [14], thyroidectomy was performed in one case [15] and plasma apheresis and thyroidectomy were required to treat hyperthyroidism in another case [16]. However, precise clinical courses, such as the onset of lethal arrhythmia or whether the case was diagnosed as TS, have not been described in these reports. Moreover, relationships between ARVC and hyperthyroidism were not considered in these reports. Taken together, the precise mechanism by which hyperthyroidism induces arrhythmia in patients with ARVC remains to be elucidated.

We considered that the patient suffered from both-sided heart failure, and the symptoms could have been inconsistent with typical acute left-sided heart failure symptoms observed in TS. Since the function of the right ventricle was originally reduced by ARVC, right ventricular dysfunction may be more predominant. Dilated cardiomyopathy-like features induced by thyrotoxicosis were reported as rare cardiac complications [5]. It may be partly due to tachycardia-induced cardiomyopathy [17]. This patient did not have a dilated left ventricle and had a low cardiac output. We speculate that due to compression by the dilated right ventricle, the dilation of the left ventricle was not apparent. Furthermore, due to the earlier clinical manifestation of right ventricular insufficiency, left ventricular dilatation may not have been apparent at admission. The left ventricular dilatation might have become apparent with time if the treatment for heart failure and TS was not initiated.

In this case, the free T3 level decreased from 26.0 pg/mL on day 2 to 16.4 pg/mL on day 5 (Figure 3). Amiodarone was started on day 3 following admission, and the free T3 level decreased possibly due to amiodarone administration as reported in a previous case [18]. Amiodarone has various effects on thyroid function, and thyroid dysfunction associated with long-term amiodarone administration is hypothyroidism or destructive thyroiditis [13]. However, the short-term effect of amiodarone treatment on thyroid function is yet to be determined. Amiodarone could decrease T3 production or thyroid hormone-related genes and may have a direct toxic effect on thyroid follicular cells as intrinsic effects [19, 20]. Moreover, amiodarone contains large amounts of iodine, and approximately 3% of its mass is actually released into the circulation as free iodine through drug metabolism [21]. Intravenous administration of amiodarone is effective for the emergency treatment of VT, and the total amount of amiodarone is particularly high on the first 2 days of initiation. In our case, 600 mg of amiodarone was administered both on the first and the second days, respectively. Consequently, it was calculated that 18 mg of free iodine was released both on the first and second days of amiodarone administration. KI (50 mg) contains approximately 38 mg of iodine and is frequently used with MMI or propylthiouracil (PTU) to treat Graves' disease. Taken together, we considered that the amount of iodine could be sufficient to decrease thyroid hormone levels as well as intrinsic effects in a short-term treatment of amiodarone. As a previous report, amiodarone might be beneficial for hyperthyroidism-induced VT among patients with Graves' disease for at least a short time [18].

The daily dose of hydrocortisone and MMI for treatment of TS were both less than their recommended doses (300 mg/day intravenous hydrocortisone and 60 mg/day oral MMI) [3]. Since a single and initial administration of 50 mg hydrocortisone and 15 mg oral MMI in addition to 100 mg KI was effective for controlling lethal arrhythmia, we considered that 150 mg/day intravenous hydrocortisone and 30 mg/day oral MMI were enough to treat his TS. Actually, his general condition improved and serum thyroid hormone levels decreased thereafter. The previous guideline stated that corticosteroid overdosing may cause unfavorable hyperglycemia and worsening of general condition; therefore, the dose needs to be carefully determined for individual patients [3]. The recommended dose of oral MMI is 60 mg/day for TS, which is the maximum dose for the treatment of Graves' disease approved by the Ministry of Health, Labor and Welfare of Japan. When large doses of MMI are initiated, the patient should be carefully monitored for potential side effects, which are dose dependent. Agranulocytosis is a rare but life-threatening complication of MMI or PTU treatment in 0.1%–0.5% of patients [22]. Agranulocytosis also shows dose dependency and rarely occurs at low doses of MMI, such as 5–10 mg/day [23]. Genetic predisposition, such as human leukocyte antigentype, has been shown to be associated with the risk of agranulocytosis as well as high dose of antithyroid drugs [24]. Previous reports showing the relationships between ARVC or PKP2 and agranulocytosis have not been identified; thus, we considered that the patient reported in the present study incidentally developed agranulocytosis due to MMI.

Total thyroidectomy was selected for the treatment of Graves' disease instead of radioiodine therapy after the onset of agranulocytosis induced by MMI for the following reasons: (1) KI and amiodarone, which include iodine, should be ceased around radioiodine therapy since there would be decreased uptake of radioactive iodine in the thyroid. The relapse of hyperthyroidism or a decrease in the serum amiodarone concentration could initiate the recurrence of lethal arrhythmia; (2) there is a possibility of elevation of serum levels of thyroid hormone after radioiodine therapy, which could lead to a TS [3]; and (3) repeated radioiodine therapy treatments could be required to control thyroid function for Graves' disease [25]. In particular, uptake of radioactive iodine in the patient's thyroid was expected to decrease due to amiodarone, which could accumulate in the thyroid over a long period regardless of the serum concentration [12]. Hence, single administration of radioactive iodine might be insufficient due to decreased uptake in this case. Therefore, total thyroidectomy was considered to be preferable to radioiodine therapy in this case. Treatment of Graves' disease should be carefully considered in each case when agranulocytosis is induced by antithyroid drugs.

## 4. Conclusion

Herein, we report the first English report of a patient with ARVC who had a refractory VT induced by TS due to Graves' disease. Furthermore, this patient suffered from biventricular heart failure. The treatment of TS was indeed effective for arrhythmia and heart failure. Clinicians should consider hyperthyroidism as a cause of refractory arrhythmia or biventricular heart failure among patients with ARVC.

## Figures and Tables

**Table 1:** Laboratory data at 6 months before the first admission, on admission, and at the time of discharge for thyroid storm.

Laboratory data	Six months before first admission	First admission for TS	Discharge for TS	Normal range
TP	6.7	6.6	6.0	6.7–8.3 g/dL
Alb	3.9	3.8	3.4	4.0–5.0 g/dL
T-bil	3.1	4.8	2.3	0.3–1.2 mg/dL
AST	31	37	18	13–33 U/L
ALT	35	25	27	8–42 U/L
LDH	209	250	160	119–229 U/L
*γ*-GTP	59	93	219	10–47 U/L
ALP	413	483	342	115–359 U/L
CPK	71	277	42	62–287 U/L
CPK-MB		7		≤25 U/L
Troponin T		0.015		≤0.014 ng/mL
BUN	12	21	13	8.0–22.0 mg/dL
Cre	0.80	0.80	0.83	0.60–1.10 mg/dL
Na	145	142	140	138–146 mEq/L
K	3.9	4.4	3.8	3.6–4.9 mEq/L
Cl	107	107	102	99–109 mEq/L
BNP	197.3	305.3	286.6	<18.4 pg/ml
WBC	6280	5800	5990	4000–9000/*μ*L
PLT	25.0	19.3	19.7	15–35 × 10^4^/*μ*L
CRP		0.21		<0.3 mg/dL
PT-INR		1.47		0.90–1.47
Antithrombin		75.0		80–120%
Fibrinogen		273		150–340 mg/dL
FDP		4		<5 *μ*g/ml
D-dimer		1.2		<1.0 *μ*g/ml
T-CHO	111	77	132	128–219 mg/dL
Triglyceride	105	41	66	30–149 mg/dL
HDL-C		26	46	40–96 mg/dL
LDL-C		39	70	<140 mg/dL

TS: thyroid storm; TP: total protein; Alb: albumin; T-bil: total bilirubin; AST: aspartate aminotransferase; ALT: alanine aminotransferase; LDH: lactate dehydrogenase; *γ*-GTP: gamma-glutamyl transpeptidase; ALP: alkaline phosphatase; CPK: creatine phosphokinase; BUN: blood urea nitrogen; Cre: creatinine; Na: sodium; K: potassium; Cl: chloride; BNP: brain natriuretic peptide; WBC: white blood cell; PLT: platelet count; CRP: C-reactive protein; PT-INR: prothrombin time international normalized ratio; FDP: fibrin/fibrinogen degradation products; T-CHO: total cholesterol; HDL-C: high-density lipoprotein cholesterol; and LDL-C: low-density lipoprotein cholesterol.

## Data Availability

The clinical data of this case report are available in the archives of our hospital.
